# Upper gastrointestinal bleeding due to hepatic artery aneurysm: Case report and literature review

**DOI:** 10.1016/j.ijscr.2020.08.045

**Published:** 2020-08-29

**Authors:** Laura Alonso-Lamberti Rizo, Carlos Bustamante Recuenco, Julián Cuesta Pérez, José Luis Ramos Rodríguez, Andrea Salazar Carrasco, Ainhoa Valle Rubio, Virginia Jiménez Carneros, Francisco Javier Jiménez Miramón, José María Jover Navalón

**Affiliations:** Hospital Universitario de Getafe, Carretera Madrid-Toledo Km 12.5, 28905, Madrid, Spain

**Keywords:** Hepatic aneurysm, Hepatic artery, Upper gastrointestinal bleeding, Embolization

## Abstract

**Introduction:**

Aneurysm of the hepatic artery is most of the time a rare and asymptomatic pathology, but in case of complication it shows high morbidity and mortality requiring in many cases an urgent treatment.

**Presentation of case:**

A 92-year-old male presented at the emergency department with high gastrointestinal bleeding and abdominal pain. Gastroscopy showed a submucosal lesion with active bleeding that was controlled through this approach. The study was expanded with a CT angiogram and a complicated hepatic aneurysm with duodenal fistulization was observed.

**Discussion:**

After reviewing the case, surgical treatment is proposed as the first option but it is rejected by the patient. Thus, aneurysm embolization with coils and thrombin is performed, without further complications.

**Conclusion:**

Selective embolization of the hepatic artery aneurysm is a therapeutic alternative in cases that implies large comorbidities, being an increasingly used technique.

## Highlights

• Hepatic artery aneurysm is an uncommon and asymptomatic disease, but in case of complication requires an urgent treatment.
• Symptomatic hepatic artery aneurysms and larger than 2 cm are indications for intervention.
• CT angiogram is the recommended technique for the diagnosis of this pathology.
• Percoutaneous embolization is an effective alternative in cases that implies large comorbidities and has become very popular.

## 1 Introduction

Hepatic artery aneurysm (HAA) is an uncommon and generally asymptomatic disease, affecting approximately 0.002% of the population [1]. Among visceral aneurysms, it is the second most frequent after splenic artery aneurysms [2]. The majority of the aneurysms are diagnosed incidentally on imaging tests, being the CT angiogram the diagnostic test of choice [3]. When symptomatic, they usually present with abdominal pain, upper gastrointestinal (GI) bleeding and/or jaundice. Surgical repair and endovascular treatment are the two therapeutic options available at present. The size of the aneurysm, its anatomical characteristics and the clinical status of the patient will ultimately determine the decision between both options [4].

We present the case of a 92-year-old man with an upper GI bleeding secondary to an hepatic artery aneurysm with duodenal fistula. This work has been reported in line with the SCARE criteria [5].

## 2 Case report

A 92-year-old male patient with a personal history of paroxysmal atrial fibrillation, colonic angiodysplasia and aneurysm of the right hepatic artery (34 mm) incidentally diagnosed 6 years ago by ultrasound during the evaluation of right upper quadrant abdominal pain. Given the age of the patient and the lack of symptoms, it was decided not to perform any therapeutic actions, so a closed follow-up was effectuated by the Angiology and Vascular Surgery Service of our centre.

The patient was admitted to the emergency department with abdominal pain, hematemesis and melaena as well as 30-min evolution hemodynamic instability. After resuscitation, an upper digestive endoscopy was performed, reporting a 20 mm round lesion on the anterior side of the duodenal bulb with submucosal compromise and a visible vessel with a clot attached and jet bleeding; initially suspicious of gastrointestinal stromal tumor (GIST). Bleeding control was achieved by performing pharmacological sclerosis with 3cc adrenaline and 5cc ethoxysclerol.

**Fig. 1:**
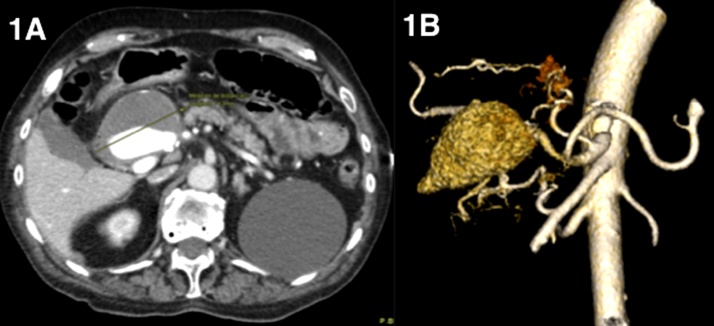
A) Right hepatic artery aneurysm, partially thrombosed. B) 3D vascular reconstruction.

Subsequently, a CT angiogram was performed to assess the submucosal lesión and rule out other possible causes. This test showed and abscence of active bleeding and an increase in the size of the previously known hepatic aneurysm (74 mm) (Fig. 1A). The aneurysm had a fusiform morphology and encompassed the right hepatic and gastroduodenal arteries, and its growth had caused an erosion of the duodenum wall that eventually produced and arterio-eneteric fistula. The patient presented an anatomical vascular variation, with an aberrant left hepatic artery originating from the left gastric artery (Fig. 1B).

**Fig. 2:**
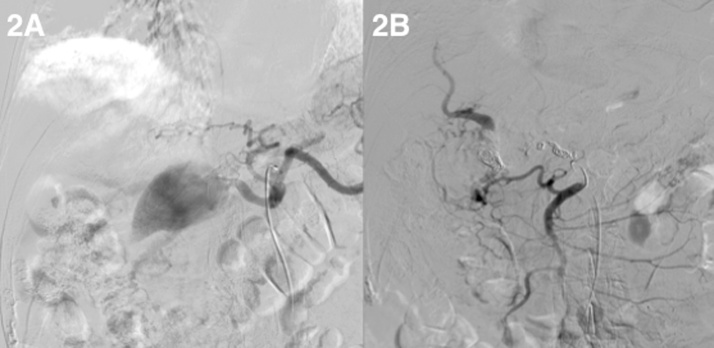
A) Visualisation of the right hepatic artery aneurysm. B) Control X ray during embolization procedure.

The case was reviewed in a multidisciplinary session and given the findings of the complementary tests, surgical treatment was offered to the patient, which he rejected assuming the poor prognosis of conservative treatment in the event of a new bleeding episode. The following day, due to the characteristics of the lesion and previous comorbidities, endovascular treatment was offered as an alternative therapy. This time the patient accepted, so embolization of the aneurysm with Interlock® coils (Boston Scientific, Natick, Massachusets, U.S.A.) and Tisseel® thrombin (Baxter International, Deerfield Illinois, U.S.A.) was perfomed under sedation. The procedure was successful and no additional sessions were needed (Fig. 2).

The patient was discharged fifteen days after admission, maintaining an outpatient follow-up. He has not present new bleeding episodes or other complications derived from the procedure thus far.

## 3 Discussion

The HAA is more frequent in men during the sixth decade of life. They are mainly associated with atherosclerosis and connective tissue disorders [6]. Its most common location is extrahepatic, meeting the following frequency: 60% common hepatic artery, 30% right hepatic artery and 5% left hepatic artery. Aneurysm rupture is the first clinical manifestation in up to 80% of cases, associating high mortality (35–85%) [7].

Among the main risk factors for rupture are the non-atherosclerotic origin and the presence of multiple aneurysms. The rupture of the aneurysm to the peritoneal cavity or to the biliary system present a similar incidence, while fistulization to the digestive system is less frequent. The communication of the lumen of the aneurysm with the biliary system might help the classic Quincke’s triad to appear, which occurs in 25% of cases and consists in hemobilia, jaundice and abdominal pain (25%) [8]. Regarding image tests, the Eco-Doppler provides information on the hepatic flow, origin and dimensions of the aneurysm, suggesting the initial diagnosis. However, it has a low sensitivity to detect small aneurysms, fact that limits its usefulness [9]. Traditionally, arteriography has been considered the gold standard in the diagnosis of this pathology. Nevertheless, over the years it has been replaced by a less invasive imaging test: the CT angiogram. Its availability, equal precisión and low complication rate make it the test of choice in this field at present [10].

Our patient presented signs of upper GI bleeding and abdominal pain, these being common to various gastrointestinal diseases. The arterio-intestinal fistula should always be taken into account as a posible diagnosis in this clinical scenario, especially in cases with previously known aneurysms. Submucosal abnormalities in gastrocopy suggested a GIST tumor in the first instance, although it is not uncommon for complicated aneurysms to have a similar endoscopic appearance to these tumors [11]. Abdominopelvic CT angiogram was performed to complete the study and obtain a more accurate diagnosis, showing the growth of the aneurysm and its fistulization to the duodenum.

Regarding the treatment of this pathology, it is important to differentiate true aneurysms from pseudoaneurysms, since the pseudo ones always require treatment due to their high bleeding risk, regardless of its size [12]. Depending on the characteristics of the aneurysm, conservative, endovascular or surgical treatment can be performed. Due to the risk of rupture, it is indicated to treat symptomatic hepatic aneurysms and those larger than 2 cm, although some authors also recommend the treatment of multiple aneurysms and those associated with polyarteritis nodosa [13].

Surgical treatments include resection with anastomosis, excision and ligation of the anuerysm, aneurysmorrhaphy and bypass. Endovascular techniques consist in percoutaneous embolization or stent graft placement. Traditionally, vascular bypass constituted the surgical treatment of choice as aneurysm ligation was reserved for high-risk cases. Percoutaneous embolization is an increasingly popular choice of treatment as it is an effective, minimally invasive procedure, and leads to an earlier recovery and discharge. It is not a complication-free technique, as incomplete aneuryms occlusions, liver necrosis and abscesses or secondary sepsis can take place, but these events ocurr infrequently [14]. It is important to remember that the superiority of one treatment over another (surgical vs. percoutaneous) has not been proven, considering that the decision must be individualized according to the characteristics of the aneurysm and the patient's age and comorbidities [15]. Our patient had an aberrant left hepatic artery and the aneurysm was located in the right one, these together with his advanced age and comorb diseases led to the decision to perform a percoutaneous embolization.

Vascular anatomy is crucial to decide the treatment. For embolization, there must be collateral circulation, patency of the portal vein and separation between the lesion and the gastroduodenal artery (GDA) [4]. Aneurysms that exclusively affect the common hepatic artery can be excluded if hepatic vascularization through the GDA is maintained. The location of the aneurysm in regard to the forementioned artery has an impact on the technique as well: if the aneurysm is located proximally, embolization is preferred; on the other hand, if it is distal, resection with subsequent reconstruction will be indicated [16]. In our case, we chose to embolize the aneurysm since the placement of stents was not recommended due to the risk of infection derived from the duodenal fistula [17]. The procedure was performed with coils and thrombin, being the aneurysm completely occluded. As an incident, highlight the accidental occlusion of the origin of the GDA during the procedure, as it was very close to the aneurysm sac. Such possibility had been previously contemplated; that is why the existence of collateral arterial flow to the territory of the GDA through the inferior pancreatoduodenal artery was confirmed before the embolization was performed. Thus, this event had no consequences for the patient’s health.

## 4 Conclusion

Duodenal fistula secondary to hepatic artery aneurysm is a rare cause of upper GI bleeding. Thus, it requires a high index of suspicion since it can associate high morbidity and mortality. Selective embolization angiography is an expanding technique that is increasingly being performed compared to surgery. Its high effectiveness and low complication rate justify its consideration as a therapeutic option in daily practice, especially in patients with numerous comorbidities.

## Funding

The authors didn’t receive any financial support for the research, authorship, and/or publication of this article.

## Ethical approval

Exception from ethical approval-case report only, consent from the patient provided at request.

## Consent

For this case report, patient accepted verbally and later signed an informed consent both for the report and the exhibits attached. The images in use are critical for the good understanding of this case, therefore for scientific purposes.

## Author contribution

Mrs. Alonso-Lamberti Rizo L.: study desing. data analysis and interpretation, writing and submission of the paper.

Mr. Bustamante Recuenco C.: study desing. data analysis and interpretation, writing and submission of the paper.

Mr. Cuesta Pérez J.: visualization.

Dr. Ramos Rodríguez JL.: interpretation of data and submission of the paper.

Mrs. Salazar Carrasco A.: interpretation of data.

Mrs. Valle Rubio A: interpretation of data.

Mrs. Jiménez Carneros V.: interpretation of data.

Dr. Jiménez Miramón FJ.: interpretation of data.

Dr. Jover Navalón JM.: interpretation of data.

## Registration of research studies

NA.

## Guarantor

Alonso-Lamberti Rizo L.

Bustamante Recuenco C.

Ramos Rodriguez JL.

## Provenance and peer review

Not commissioned, externally peer-reviewed.

## Declaration of Competing Interest

The authors declare no conflict of interest.
